# Identification of lactylation gene CALML5 and its correlated lncRNAs in cutaneous melanoma by machine learning

**DOI:** 10.1097/MD.0000000000035999

**Published:** 2023-11-24

**Authors:** Hailiang Feng, Wei Chen, Chen Zhang

**Affiliations:** a Department of Stomatology, Linping Campus, Second Affiliated Hospital, Zhejiang University School of Medicine, Hangzhou, China; b Department of Emergency Surgery, Linping Campus, Second Affiliated Hospital, Zhejiang University School of Medicine, Hangzhou, China.

**Keywords:** CALML5, cutaneous melanoma, immune microenvironment, lactylation, lncRNA

## Abstract

As a product of glycolysis, lactate contributes to cancer proliferation, immunosuppression, and metastasis via histone lactylation. However, the relationship between cutaneous melanoma (CM) and lactylation-associated genes and lncRNAs has remained unclear. In this study, 4 mechanism learning algorithms and integrated bioinformatic analyses were employed to identify the core lactylation-associated genes and lncRNAs. Subsequently, 2 risk signatures based on the hub lactylation-associated genes and lncRNAs were constructed for CM patients. As a result, CALML5 was identified as a core lactylation-associated gene in CM, and its expression was found to be associated with patients survival and immune infiltration, suggesting its relevance as a potential therapeutic target. Additionally, this study provided clarification on hub CALML5-associated lncRNAs in CM, offering insights into their roles in the disease. Meanwhile, 2 identified risk signatures were both strongly linked to the prognosis and cancer growth of CM, underscoring their potential as valuable prognostic indicators. Furthermore, mechanistic analyses suggested a significant association between the risk signature and the immune microenvironment in CM, highlighting potential immune-related implications in disease progression. In conclusion, we propose that lactylation-associated genes and lncRNAs hold promise as potential targets in CM. Moreover, our findings revealed a significant correlation between lactylation and the immune microenvironment, providing crucial insights for guiding individualized treatment strategies in CM.

## 1. Introduction

Cutaneous melanoma (CM), a malignant tumor,^[1]^ ranks fifth among prevalent cancers in male patients and 6th in female patients, according to the 2020 cancer statistics.^[2]^ CM is considered curable by extended surgical resection at the early stage, with a 5-year survival probability of approximately 40% to 50%.^[3]^ Unfortunately, the majority of CM patients are diagnosed with advanced metastatic CM, leading to a significant reduction in their 5-year survival prognosis, which stands at 19%.^[4,5]^ Personalized therapy and prognostic prediction for CM necessitate the identification and utilization of specific biomarkers. However, the current list of biomarkers identified with clinical significance for CM is insufficient.^[6]^ Therefore, the identification and screening of novel biomarkers, along with the construction of prognostic signatures capable of accurately predicting a patient’s condition, become urgent priorities.

As the final product of the Warburg effect which identifies excessive lactate accumulation and abnormal anaerobic glycolysis,^[7]^ lactate was previously described only as a metabolite of glycolysis. However, recent studies have proved that lactate is not just an energy material, but also participate in intercellular communication and immunomodulatory process.^[8]^ Meanwhile, it is widely present in cancer samples.^[9,10]^ Undergoing abnormal metabolism, cancer cells continue to intake glucose and make various lactate, these accumulated lactates will be exported to the extracellular environment and formed the acidic tumor microenvironment.^[11]^ In addition, histone lysines, which is the key mechanism for lactates to fulfill its duties, will also be modified through lactylation, and then participate in several crucial biological processes, like tumor proliferation^[12]^ and macrophage polarization.^[13]^ Although the modification of lactylation has received widespread attention, the accurate mechanisms responsible for the potential role of lactylation in tumor proliferation remain unclear, and the association between lactylation and CM has yet to be explored.

Recent progress in bioinformatic analyses has enabled researchers to identify numerous biomarkers that are specific to various diseases. Despite of that, recent progress in bioinformatic analyses has enabled researchers to identify numerous disease-specific biomarkers. However, there is a lack of identifiable genes associated with lactylation and the prognosis/progression of CM. Thus, we conducted a screening of hub lactylation-associated genes and lncRNAs significantly associated with CM patient prognosis. Subsequently, based on the expressions of hub lactylation-associated genes and core lncRNAs, we constructed 2 risk signatures for CM patients. The clinical significance and prognostic role of these risk signatures were also evaluated. Furthermore, we observed close connections between lactylation-associated genes and the risk signature with the immune microenvironment. In summary, this study is the first to identify the role of lactylation-associated genes and lncRNAs in CM, presenting a promising target for predicting prognosis and clinical outcomes in CM patients.

## 2. Materials and methods

### 2.1. Acquisition of raw data

RNA sequencing datasets of 471 CM patients with clinical information were collected from the cancer genome atlas (TCGA, https://portal.gdc.cancer.gov/) database. Additionally, to address the lack of control samples (only 1 normal skin) in the TCGA database, 812 normal skin samples were obtained from the genotype-tissue expression (http://commonfund.nih.gov/GTEx/) database. To facilitate comparative analysis of gene expression data obtained from different platforms, the “limma” R package was employed to normalize the data and convert it into a single matrix. Subsequently, the “sva” R package was used to remove batch effects. Based on prior research,^[14]^ 327 lactylation-related genes were identified through screening. For external validation, additional CM samples were collected from the GEO-GSE65904 cohort. CNV data of CM patients were also obtained from the TCGA database. The search of all public databases in this study was conducted in compliance with the relevant guidelines. As it did not involve human or animal subjects, ethical approval was not required.

### 2.2. Machine learning model construction

Firstly, univariate Cox regression analysis was performed on TCGA-CM patients to identify the prognostic lactylation-associated genes. Thereafter, these genes were further integrated into the least absolute shrinkage and selection operator (LASSO) analysis to identify the candidate lactylation-associated genes in CM. Candidate lactylation-associated genes were subjected to construct 4 different machine learning models, including generalized linear models, random forest, support vector machine, and extreme gradient boosting, using R packets “xgboost,” “random- Forest,” “dalex,” and “caret,” respectively. These 4 machine learning tasks were conducted using the “kernlab” R package. To assess the accuracy of these mechanism models, residual analysis was applied to the samples, and the reverse cumulative distribution of residuals was plotted to evaluate the models. The receiver operating characteristic (ROC) curves of the 4 machine learning models were plotted using the “pROC” R package. To identify key genes for CM patients, each candidate lactylation-associated gene in the models was assigned an importance score. Based on the results of the 4 machine learning models, the hub lactylation-associated gene in CM was identified, and KM survival analysis, along with ROC analysis, was performed for the core gene.

### 2.3. Functional enrichment analyses

The database for annotation, visualization, and integrated discovery (version 6.8) was utilized for gene ontology analysis to determine the biological functions of candidate lactylation-associated genes. Gene ontology enrichment analyses were conducted for cellular components, molecular function, and biological processes. Thereafter, GSEA (gene set enrichment analysis) and GSVA (gene set variation analysis) analyses were performed to investigate the potential mechanism of the hub-identified lactylation-associated gene CALML5. GSEA was conducted on the gene expression matrix using the “c2.cp.kegg.symbols.gmt” sets to detect Kyoto encyclopedia of genes and genomes pathways. According to the GSVA score matrix, gene-level changes were transformed into pathway-level ones using the “GSVA” R package, ultimately enabling the evaluation of potential biological functions. Statistical significance was defined as false discovery rate and *P* values < .05.

### 2.4. Screening for overlapping hub lncRNAs

The identification of hub lncRNAs involved performing Pearson correlation analysis (|R2| > 0.15, *P* < .05) to assess the connections between CM and CALML5-associated lncRNAs, followed by univariate Cox regression analysis, differential expression analysis, and WGCNA (weighted gene co-expression network analysis). The cutoff criterion of *P* < .05 was utilized in the univariate Cox regression analysis. The R package “limma” was utilized to conduct differential expression analysis for CALML5-associated lncRNAs in CM, employing criteria of |log fold change| > 1 and *P* < .05. With the assistance of the R package “WGCNA,” we established the co-expression network linking CALML5-associated lncRNAs with sample modules. Based on the scale-free topological criterion ( > 0.87) and a minimum power value, the best soft threshold was determined to be 3. Later, the weighted adjacency matrix was used to generate a topological overlap matrix, and hierarchical clustering was applied to construct dendrograms based on the topological overlap matrix. To control the number of modules generated, the main parameters were defined with a min Module Size of 10. In conclusion, CALML5-associated lncRNAs displaying notable interconnections were assigned to various patterns. The calculation of gene significance and module eigengenes was carried out for all modules. The “Venn Diagram” package was utilized to visualize the overlap of lncRNAs among WGCNA, differential expression, and univariate Cox regression analyses, and these shared lncRNAs were designated as hub lncRNAs for further investigation. The “survival” R package was utilized for KM survival analysis, and the predictive accuracy of hub lncRNAs were verified using the R package “pROC”.

### 2.5. Construction of the gene and lncRNA signatures

Gene and lncRNA risk signatures were generated based on the expression of 8 candidate lactylation-associated genes and 8 identified hub CALML5-associated lncRNAs, respectively. These selected lncRNAs and genes were applied into a LASSO penalized Cox regression analysis, and the risk score of signatures were constructed using the following formula:


$$\begin{array}{l} Risk\;score=\Sigma expi*\beta i. \end{array}$$

Where expire presents the relative expression of lactylation-associated genes or hub lncRNAs i, and β is the regression coefficient. Then, based on the median value of the constructed risk score, CM patients were segregated into low- and high-risk subgroups.

The “ggplot2” and “Rtsne” packages were employed, and PCA and t-SNE were conducted to investigate the distribution of risk subgroups. Then, Cox regression and KM survival analyses were employed to assess and compare the prognostic ability. Next, the “timeROC” and “cliROC” packages were applied to calculate the accuracy of this risk signatures for prediction. We utilized the “RMS” package to construct a nomogram that utilized the risk scores of the gene signature for predicting CM patients outcomes, and the accuracy and discrimination were subsequently evaluated through decision curve analysis.

### 2.6. Immune correlation analysis

Firstly, CIBERSORT analysis was conducted to assess the relationship between the expression of lactylation-associated genes and immune cell infiltration. Thereafter, single-sample GSEA was performed to explore the discernible differences in immune cell infiltration and immune functions among CM risk subgroups. The relationship between the scores of immune-associated factors (including stromal and immune scores) and the risk signature was evaluated by Spearman correlation analysis. By referring to the potential immune checkpoints identified in a previous study, we ascertained the link between the risk signature and immune-associated genes.^[15]^

## 3. Results

### 3.1. Risk signature of lactylation-associated genes for CM patients

**Figure 1.**
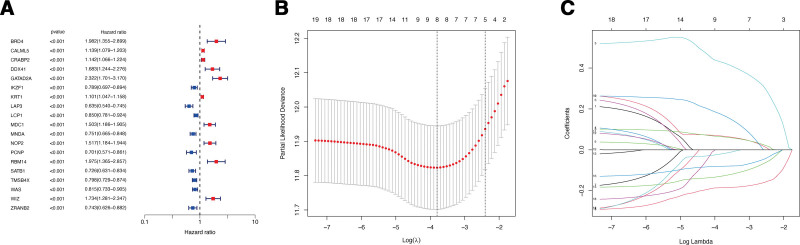
Identification of candidate lactylation-associated gene in CM. (A) The univariate Cox regression analysis. (B–C) LASSO analysis to determine factors and construct the model. CM = cutaneous melanoma, LASSO = least absolute shrinkage and selection operator.

**Figure 2.**
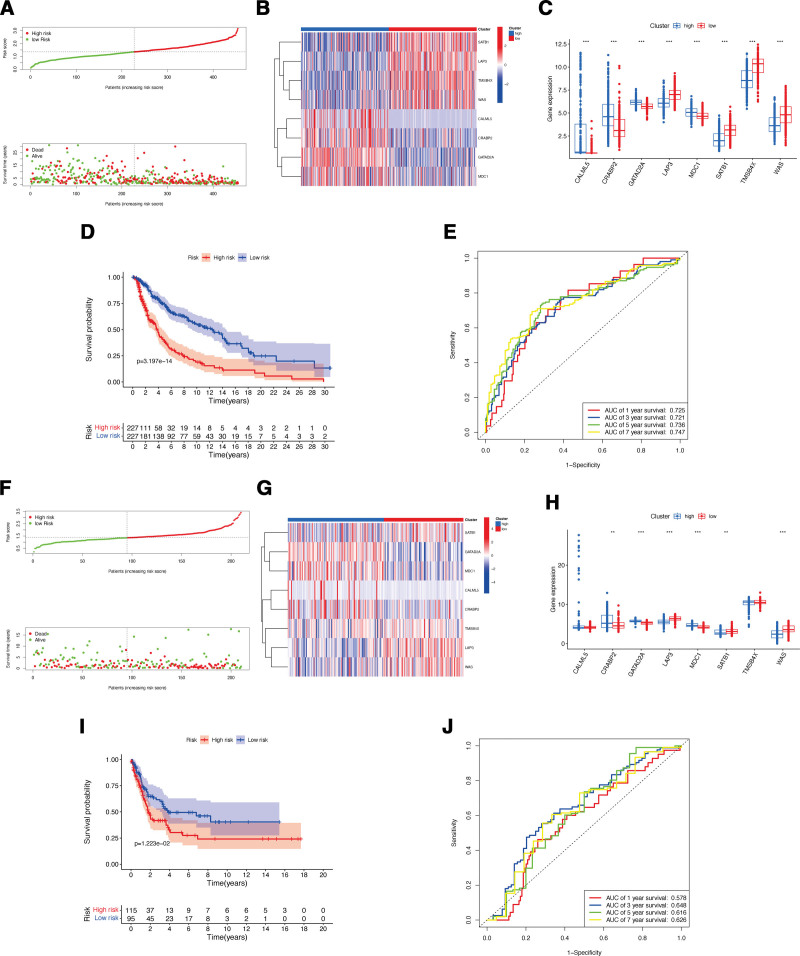
Construction of lactylation-associated genes risk signature. (A) The risk score distributions in the TCGA-CM set. (B–C) The differential expression of lactylation-associated genes in the risk subgroups of TCGA-CM set. (D–E) The survival status of risk signature in the TCGA-CM set. (F) The risk score distributions in the GSE65904 set. (G–H) The differential expression of lactylation-associated genes in the risk subgroups of GSE65904 set. (I–J) The survival status of risk signature in the GSE65904 set. CM = cutaneous melanoma, TCGA = the cancer genome atlas.

**Figure 3.**
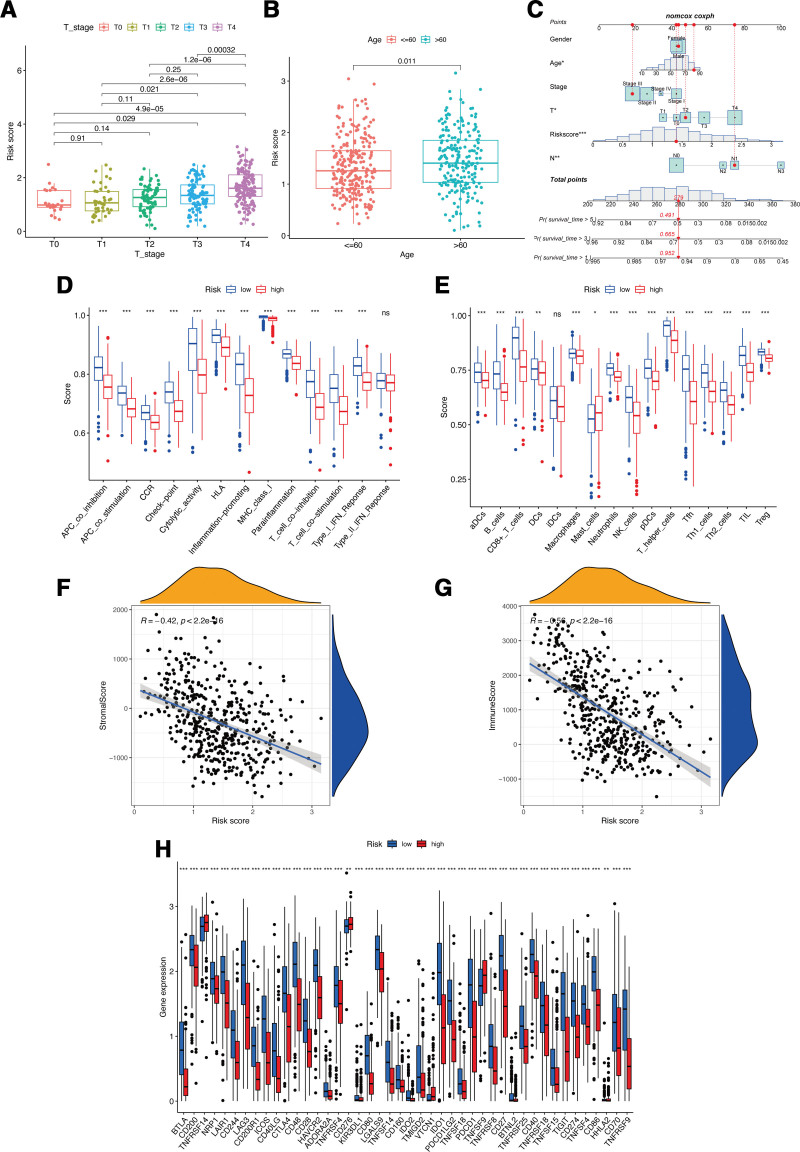
Associations between risk signature, clinicopathological factors, and immune characteristics. Correlations between risk scores and T stages (A) and ages (B). (C) Nomogram for predicting the OS of risk scores. Boxplots of scores of immune-associated functions (D) and immune cells (E) in risk signature. Associations between risk signature, stromal scores (F) and immune scores (G). (H) Expression of immune checkpoints among two risk subgroups in CM patients. CM = cutaneous melanoma, OS = overall survival.

Through the univariate Cox regression analysis with *P* < .001, 19 lactylation-associated genes significantly associated with the overall survival (OS) of CM patients were identified in TCGA patients (Fig. 1A). For the construction of the risk signature model, the LASSO analysis was applied for these 19 genes, and 8 genes (CRABP2, GATAD2A, MDC1, LAP3, SATB1, TMSB4X, CALML5, and WAS) was identified as the candidate lactylation-associated genes in CM and applied for lactylation-associated genes risk signature construction (Fig. 1B and C). According to the calculated median value of risk scores, 2 subgroups were classified as high- or low-risk score for the patients from the cohorts of TCGA-CM (Fig. 2A) and GSE65904 (Fig. 2F). During the investigation of the connection between lactylation-associated genes and risk subgroups, it was observed that genes CRABP2, GATAD2A, and MDC1 were higher expressed, and genes LAP3, SATB1, and WAS were lower expressed in high-risk subgroup in both 2 databases with significance value (Fig. 2B,C,G,H). The KM survival analysis indicated that in both TCGA and GEO cohort, the CM patients OS is negatively correlated with their risk scores (Fig. 2D and I). Additionally, in the cohort of TCGA-CM, the AUC values for the moderate predictive accuracy contained in our risk signature at 1-year, 3-year, 5-year, and 7-year follow-up are all over than 0.7 (Fig. 2E). The AUC in the cohort for validation at 1-year, 3-year, 5-year, and 7-year follow-up are 0.578, 0.648, 0.616, and 0.626 respectively (Fig. 2J). Additionally, compared to patients with lower T stage or aged< = 60, the patients with higher T stage or aged > 60 exhibited obviously higher risk scores (Fig. 3A and B). Finally, the nomogram plot of CM patients was created by incorporating the clinical characteristics and risk score (Fig. 3C), with the aim of utilizing the prognostic value of risk signature.

### 3.2. Relationship of the lactylation-associated gene risk signature with immune microenvironment

In comparison to those patients with low-risk scores, the patients with high-risk scores merely exhibited a significantly reduced all immune cell functions and subpopulations, but except mast cells (Fig. 3D and E). Next, the associations of the risk signature with immune microenvironment were further clarified, and the results showed a significantly negative connection between our constructed risk signature to stromal (Fig. 3F) and immune (Fig. 3G) scores. Within the 2 risk subgroups, immune checkpoints displayed notable differential expression, CM patients with high-risk scores showed a marked reduction in the expression of various immune-associated genes, like BTLA, CD200, NRP1, and so on, except for TNFRSF14, CD276, VTCN1, and TNFSF9 (Fig. 3H).

### 3.3. Expression and mechanism of lactylation-associated genes in CM

**Figure 4.**
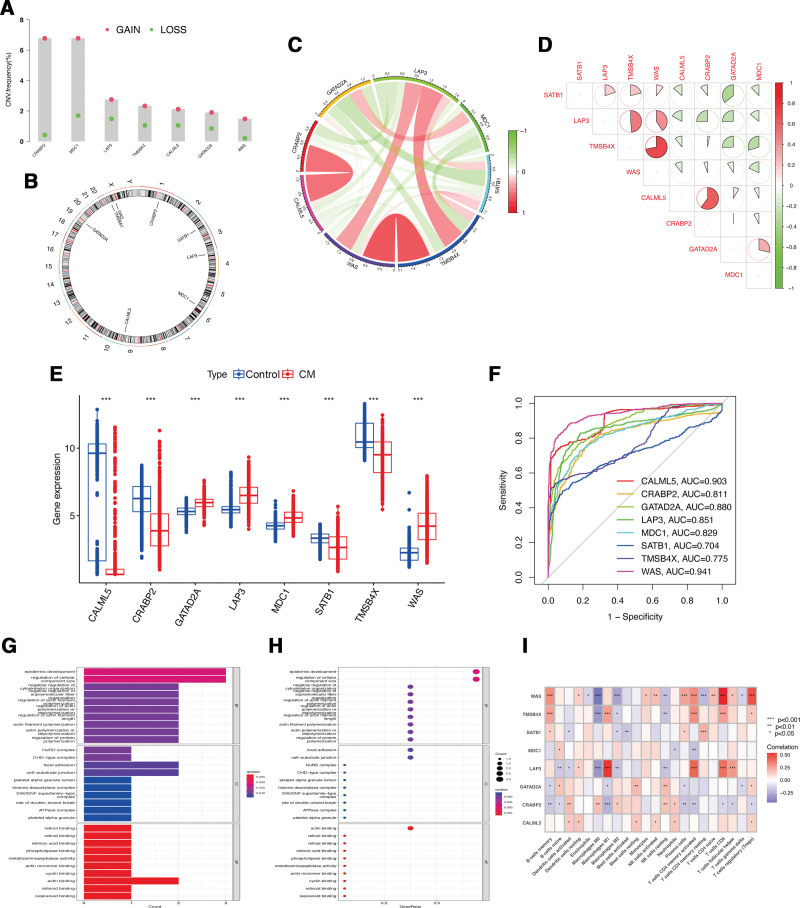
The role of candidate lactylation-associated genes in CM. (A) The CNV variation frequency of lactylation-associated genes. (B) The location of CNV alteration of lactylation-associated genes on 23 chromosomes. (C–D) The correlation between 8 lactylation-associated genes. (E) The expression difference of lactylation-associated genes between normal and CM samples. (F) ROC curve of lactylation-associated genes for CM. The GO enrichment terms of lactylation-associated genes in CC, BP, and MF are shown by a bar plot (G) and bubble chart (H). (I) Correlation heatmap depicting correlations between infiltrated immune cells and lactylation-associated genes in CM. BP = biological processes, CC = cellular components, CM = cutaneous melanoma, GO = gene ontology, MF = molecular function, ROC = receiver operating characteristic.

Investigation of somatic copy number variations (CNVs) in identified lactylation-associated genes in CM revealed a higher prevalence of CNVs in the genes WAS, GATAD2A, CALML5, TMSB4X, LAP3, MDC1, and CRABP2. Specifically, all these genes exhibited increased CNV changes (Fig. 4A). The alterations of CNVs across the respective chromosomes of identified lactylation-associated genes are depicted in Figure 4B. Interestingly, network visualization of the correlation between lactylation-associated genes showed that all genes exhibited significant associations with each other (Fig. 4C and D). The expression levels of GATAD2A, LAP3, MDC1, and WAS were all significantly evaluated in CM samples compared to normal skin tissues, whereas genes CALML5, CRABP2, SATB1, and TMSB4X were significantly lower expressed in CM (Fig. 4E). To further confirm the predictive accuracy of lactylation-associated genes in predicting the occurrence of CM, it was discovered that genes CALML5 and WAS had high predictive value with AUC > 0.9, and genes CRABP2, GATABP2, LAP3, MDC1, SATB1, and TMSB4X had moderate predictive value in CM with AUC > 0.7 (Fig. 4F).

Functional analyses were conducted based on the intersection of identified lactylation-associated genes. Regarding biological processes, the genes were mostly enriched in epidermis development, regulation of cellular component size, and negative regulation of cytoskeleton organization, et al In terms of cellular components, the genes were linked to NuRD complex, CHD-type complex, and focal adhesion, et al As for molecular function, the genes were mainly involved in binding of retinol, retinal, and retinoic acid, et al (Fig. 4G and H). To investigate the relationship of lactylation-associated genes with immune infiltration in CM, Figure 4I indicated that 8 identified lactylation-associated genes all exhibited a significant correlation with the proportions of immune cells, thereby providing evidence for the potential of lactylation-associated genes as prognostic targets for CM immunotherapy.

### 3.4. Construction of machine learning models and identification of hub lactylation-associated gene

**Figure 5.**
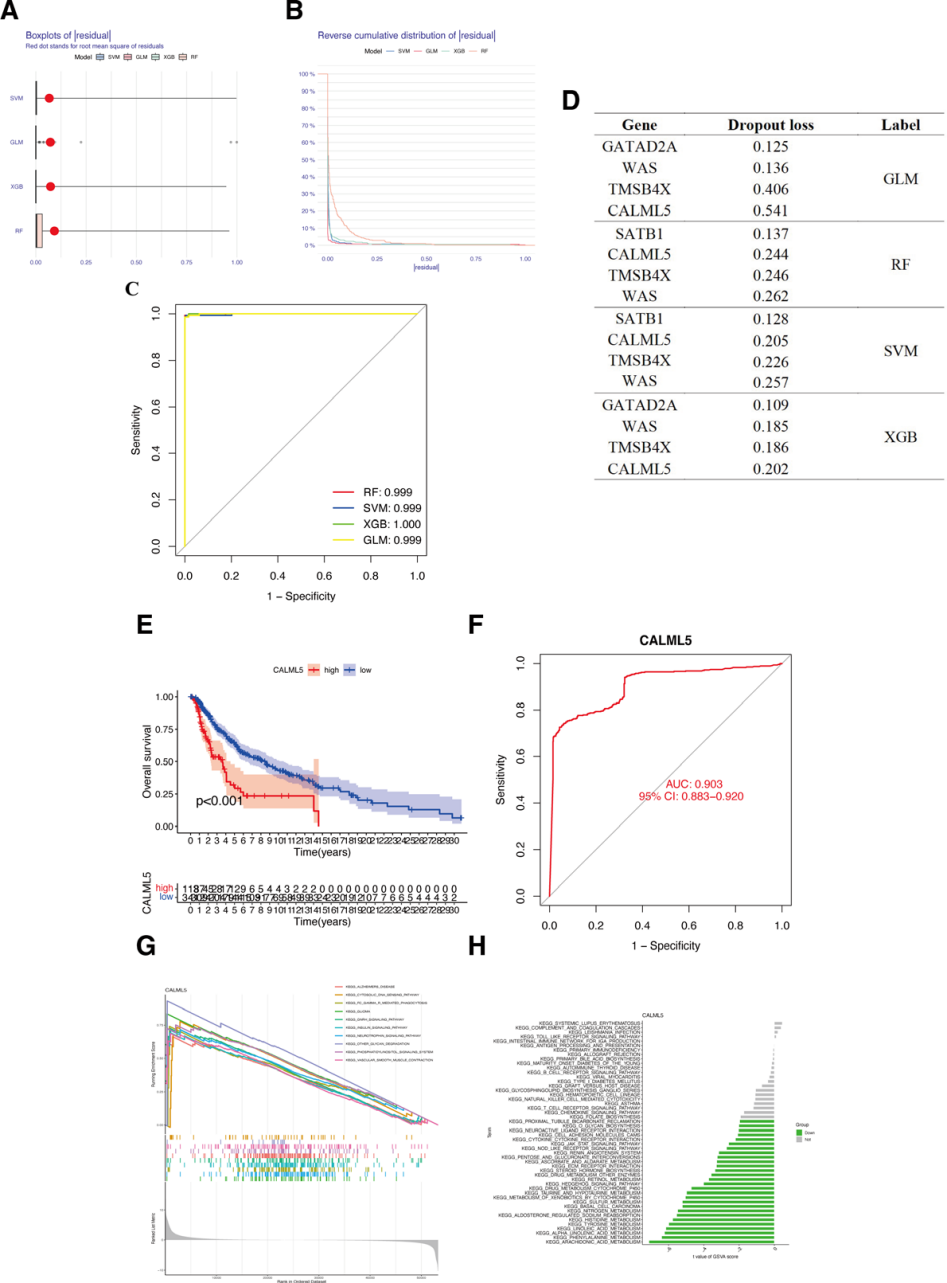
Identification of core lactylation-associated gene in CM by machine learning models. (A) Box plots of sample residuals of the four algorithms. (B) The reverse cumulative distribution map of model residuals. (C) ROC analysis of algorithms. (D) Importance score of feature genes in the model. (E) The KM curve of gene CALML5 in TCGA-CM patients. (F) ROC curve showed the prediction accuracy of CALML5 for CM. GSEA (G) and GSVA (H) analyses of CALML5. CM = cutaneous melanoma, ROC = receiver operating characteristic, TCGA = the cancer genome atlas.

In this study, to develop diagnostic models, we employed 4 different algorithms, namely extreme gradient boosting, support vector machine, random forest tree, and generalized linear models. The analyses of these models, including box plots (Fig. 5A) and reverse cumulative distribution (Fig. 5B), consistently showed small residuals between 0 and 0.1 for all 4 algorithms used. The results of ROC curves confirmed the high accuracy of the diagnostic models, as evidenced by the AUC values, which were all close to 1 for the 4 algorithms employed (Fig. 5C). In the evaluation of the importance scores of the 4 algorithms, the gene WAS and CALML5 showed the highest score (Fig. 5D). WAS is a member of WASP family which has been proved to play a virtual role in melanoma cells migration and invasion through reorganizing actin cytoskeleton.^[16,17]^ However, concerning the role of CALML5 in CM, there is currently only 1 reference indicating the differential expression of CALML5 in the CM clusters,^[18]^ its specific prognostic value and modulation mechanism in CM remains unclear. Thus, in this study, we ultimately chose CALML5 for subsequent prognostic analysis. To further confirm the role of CALML5 in CM prognosis, KM analysis indicated that the expression of CALML5 was significantly negatively connected with the OS of CM patients (Fig. 5E). CM patients in the low CALML5 expression subgroup had a significantly better prognosis. Meanwhile, the AUC values for CALML5 were 0.903, revealing that CALML5 could specifically predict CM occurrence (Fig. 5F). Moreover, we also observed tight associations between CALML5 and several pathways, including vascular smooth muscle contraction, phosphatidylinositol signaling system, arachidonic acid metabolism, T cell receptor signaling pathway, and others, from our GSVA and GSEA enrichment analysis (Fig. 5G and H).

### 3.5. Identification of hub CALML5-associated lncRNAs in CM

**Figure 6.**
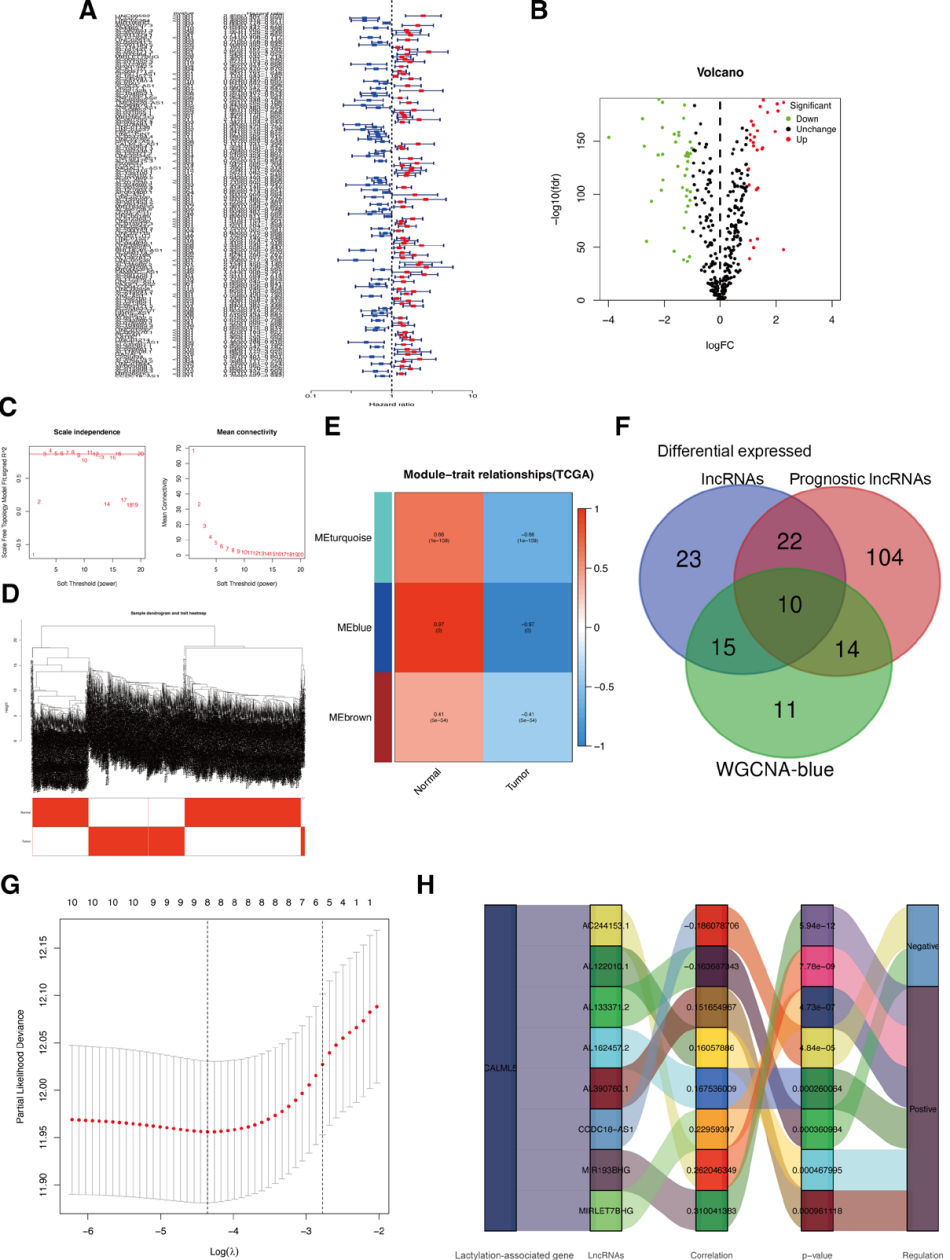
Characterization of hub CALML5-associated lncRNAs in CM. (A) Univariate Cox regression analysis showed prognostic lncRNAs. (B) Volcano plot of differentially expressed lncRNAs. (C–D) Soft-thresholding powers scale-free fit index. (E) Heatmap showing the correlation between clinical traits and gene module. (F) The Venn diagram. (G) LASSO analysis identified hub lncRNAs. (H) Correlation network of hub lncRNAs and CALML5. CM = cutaneous melanoma, LASSO = least absolute shrinkage and selection operator.

**Figure 7.**
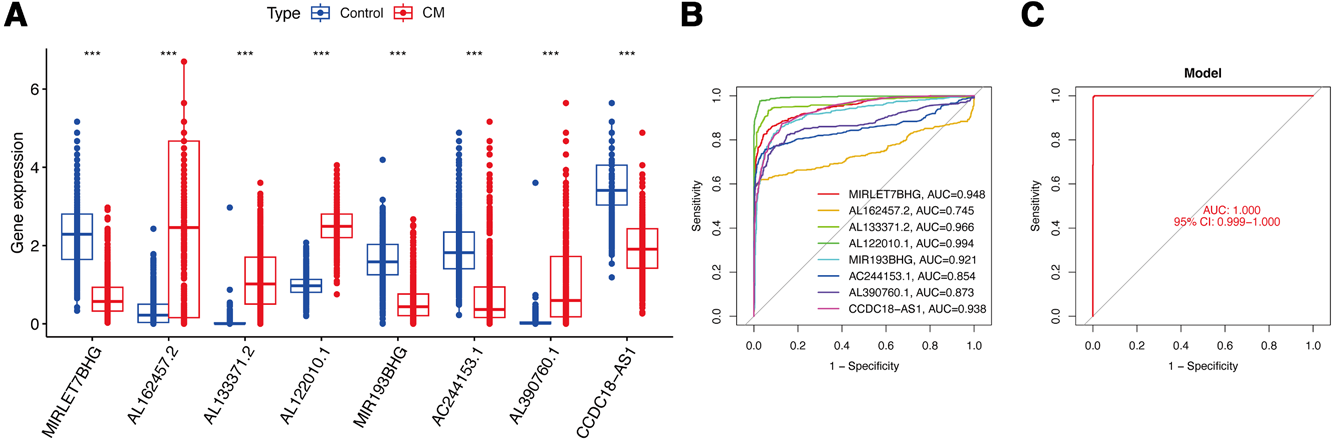
The Role of hub lncRNAs in CM. (A) Gene expression of hub lncRNAs. (B–C) ROC curve of hub lncRNAs. CM = cutaneous melanoma.

The researchers employed correlation analysis to identify 357 lncRNAs significantly correlated with hub gene CALML5, which were then utilized for further investigations. Then, 150 prognosis-associated lncRNAs (Fig. 6A) and 70 differentially expressed lncRNAs (Fig. 6B) were identified through screening. To gain deeper insights into the functional clusters linked to CM patients, WGCNA analysis was extended to include CALML5-associated lncRNAs (Fig. 6C and D). The blue module exhibited the strongest association (*R* = 0.97) between the 2 clusters, prompting the choice of lncRNAs from this module for subsequent analysis (Fig. 6E). Based on the above analyses, 10 overlapping lncRNAs were selected as candidate lncRNAs (Fig. 6F). Thereafter, LASSO analysis was applied to shrink the number of lncRNAs, and 8 hub lncRNAs (MIRLET7BHG, AL162457.2, AL122010.1, MIR193BHG, AC244153.1, AL390760.1, AL133371.2, and CCDC18-AS1) were ultimately selected for further prognostic analysis (Fig. 6G), and in Figures 6H, we can find the graphical representations of how the identified hub lncRNAs are connected to CALML5. While exploring the role of hub lncRNAs in CM, the results of the gene expression analysis for hub lncRNAs in CM revealed a significant downregulation of lncRNA MIRLET7BHG, MIR193BHG, AC244153.1, and CCDC18-AS1 in CM tissues compared to normal samples (Fig. 7A). Conversely, AL390760.1, AL122010.1, AL133371.2, and AL162457.2 were significantly upregulated in CM tissues. The ROC analysis indicated that, as individual diagnostic biomarkers, MIRLET7BHG, AL133371.2, AL122010.1, MIR193BHG, and CCDC18-AS1 exhibited an AUC of above 0.9, while AL162457.2, AC244153.1, and AL390760.1 displayed AUCs of above 0.7 (Fig. 7B). Meanwhile, when 4 hub lncRNAs were combined into a prediction model, the ROC analysis showed that the predictive accuracy of CM increased to 1 (Fig. 7C). These findings highlight the high predictive accuracy of the 3 identified lncRNAs in CM patients.

### 3.6. CALML5-associated lncRNAs risk signature value in CM clinics

**Figure 8.**
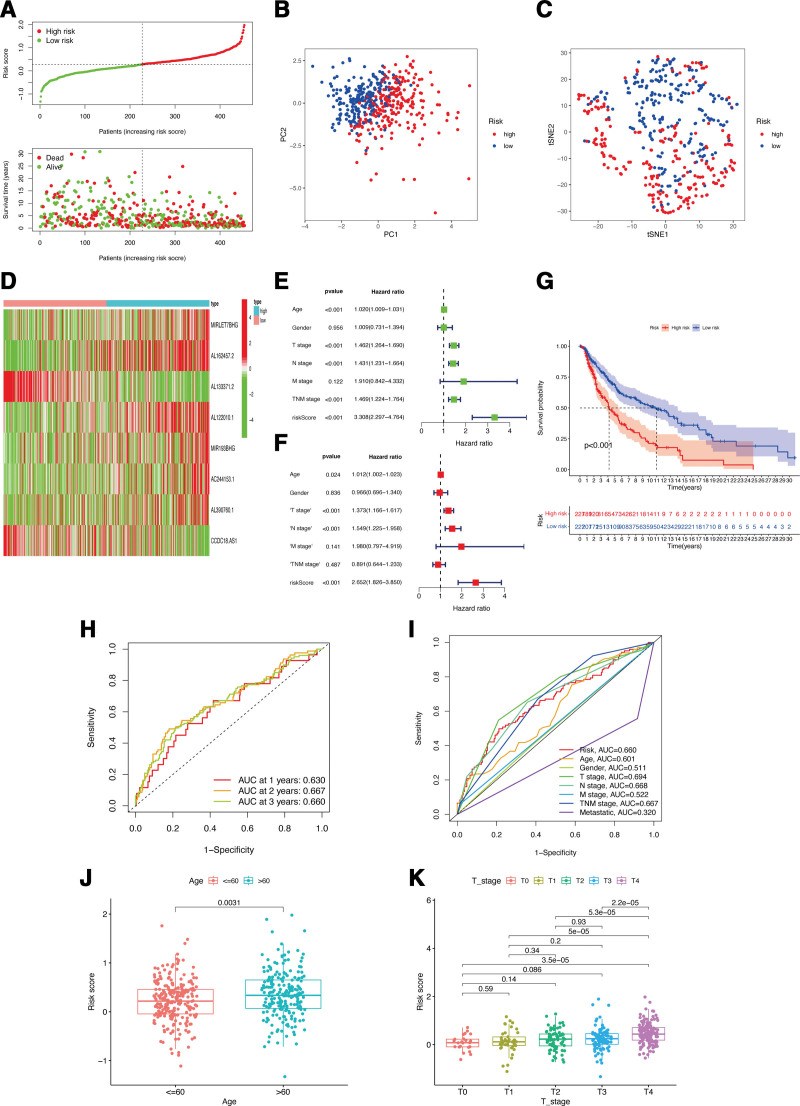
Associations between CALML5-associated lncRNAs risk signature and clinicopathological factors. Risk score distribution (A), PCA plot (B), and t-SNE (C) analysis of TCGA-CM cohort. (D) The heatmap of lncRNAs expression in two risk subgroups. Univariate (E) and multivariate Cox (F) regression of risk signature. (G) Survival curve of CM patients. Time ROC (H) and clinical ROC (I) curves to forecast OS of patients. Correlations between risk scores and ages (J), and T stage (K). CM = cutaneous melanoma, OS = overall survival, ROC = receiver operating characteristic, TCGA = the cancer genome atlas.

Based on the median value of the calculated risk scores by the identified CALML5-associated lncRNAs, CM patients were classified into 2 subgroups (Fig. 8A). Clear separation of these 2 subgroups was observed in the PCA (Fig. 8B) and t-SNE (Fig. 8C) analyses. During the investigation of the connection between hub lncRNAs and the CM signature, it was observed that lncRNAs MIRLET7BHG, AL162457.2, AL122010.1, MIR193BHG, AC244153.1, and AL390760.1 were higher expressed in the high-risk subgroup, whereas AL133371.2 and CCDC18-AS1, was lower expressed in such subgroup (Fig. 8D). Furthermore, our analysis provided evidence that the risk signature of lncRNAs could independently predict outcomes for CM patients (Fig. 8E and F). High-risk scores in CM patients were associated with a considerably reduced OS compared to low-risk scores (Fig. 8G). According to ROC analysis (Fig. 8H), the risk signature exhibited a moderate predictive accuracy at 1 (AUC = 0.630), 2 (AUC = 0.667), and 3 (AUC = 0.660) years. When contrasted with other traditional clinicopathological features, the risk signature demonstrated superior accuracy in clinical ROC curve analysis (Fig. 8I), underscoring its sensitivity and specificity in predicting CM patient survival. Moreover, compared to patients with aged ≤ 60 (Fig. 8J) or lower T stage (Fig. 8K), the patients with higher T stage or aged > 60 exhibited obviously higher risk scores.

## 4. Discussion

The application of advanced sequencing technologies in the field of biology has led to the discovery of a growing number of biomarkers that are associated with melanoma. Nonetheless, there is still a pressing need to identify biomarkers with the potential for early detection or prognostic prediction among patients with CM. The modification of lactylation is widespread in human cells and modified by glycolysis.^[19]^ It has been discovered that lactylation is connected with cancer metabolism and immune regulation.^[20]^ However, the precise involvement of lactylation in the pathogenesis and clinical behavior of CM, including its contribution to the tumorigenesis, disease progression, and metastatic spread, remains largely elusive and warrants further investigation. Herein, we identified the hub lactylation-associated gene CALML5 and its core associated lncRNAs in CM. Meanwhile, risk signatures were constructed based on the expression of identified lactylation-associated genes and lncRNAs, and their high precision for predicting the OS of CM patients was further demonstrated. In addition, we observed significant correlations of lactylation-associated genes and risk signatures with the immune microenvironment, indicating their potential utility and clinical advantages.

To elucidate the relationships between lactylation and the OS of patients with CM, we conducted a systematic analysis of lactylation-associated genes. As a result, genes WAS, GATAD2A, CALML5, TMSB4X, LAP3, MDC1, SATB1, and CRABP2 were identified as candidate lactylation-associated genes in CM. It was discovered that all these genes showed significant differential expression in CM samples compared to normal skin. Additionally, by utilizing 4 mechanism learning algorithms and ROC analysis, CALML5 exhibited a high importance score in predicting CM compared to other candidate genes, with a high predictive accuracy and significant prognostic value for CM. Based on these findings, CALML5 was identified as the core lactylation-associated gene in CM.

Calmodulin-like 5 (CALML5, also named calmodulin-like skin protein) expression is limited to the epidermis, and especially abundant in differentiated epidermis.^[21]^ Owing to its virtual role in maintaining an epidermal barrier, loss of CALML5 can promote the progression of squamous cell carcinoma.^[22]^ Recently, literatures confirmed CALML5 participates in not only the differentiation of epidermal but also differentiation, proliferation, and migration of cancer cells.^[23]^ For example, CALML5 performed as a key biomarker for lymphatic vascular infiltration in breast cancer and significant connected with breast carcinogenesis.^[24]^ Furthermore, the methylation of gene CALML5 has a high predictive capability in the poor OS of HPV-positive oropharyngeal cancer patients.^[25]^ In CM, study indicated that CALML5 was involved in the pathway of “hsa04916: Melanogenesis.”^[18]^ Mac et al^[26]^ also found that calmodulin-like protein could promote the progression of CM sample, which was is in line with our discoveries that the expression of CALML5 was negative associated with the OS of patients with CM. This finding may be related to the modulation of immune cells and pathways. Immunotherapy has been recognized as one of the most promising therapeutic approaches for improving outcomes in various types of malignant tumors.^[27]^ In this regard, we observed enrichments of CALML5 in immune-associated pathways, including T cell receptor signaling pathway, natural killer cell mediated cytotoxicity, and more. Additionally, the expression of CALML5 in CM was positively correlated with the infiltration of immune cells such as activated/resting dendritic cells, resting mast cells, activated NK cells, neutrophils, and regulatory T cells. Considering the pivotal effect of immune cells on the regulation of immune response against tumors,^[28]^ this observed correlation with immune infiltration holds significant value in predicting the OS of CM patients by CALML5. However, the functional mechanism of CALML5 with immune cells still needs further investigation.

Afterward, 8 lactylation-associated genes (CRABP2, GATAD2A, MDC1, LAP3, SATB1, TMSB4X, CALML5, and WAS) and 8 CALML5-associated lncRNAs (MIRLET7BHG, AL162457.2, AL122010.1, MIR193BHG, AC244153.1, AL390760.1, AL133371.2, and CCDC18-AS1) were utilized for constructing risk signatures, aiming to predict CM prognosis. Various methods were utilized to confirm the utility of these constructed risk signatures in predicting CM prognosis. These risk signatures were closely linked to CM OS, ages, and T stage, as observed in our study. As a clinicopathological parameter, the American Joint Committee on Cancer staging system is commonly applied in the assessment of tumors.^[29]^ In predicting CM growth and prognosis, our risk signatures showed superior accuracy compared to the TNM stage. Furthermore, a nomogram analysis confirmed the efficacy of the lactylation-associated genes risk signature in predicting CM outcomes.

Furthermore, significant connections of lactylation-associated genes risk signature to immune microenvironment were confirmed by several methods, the link to immune processes indicates its potential as a prognostic indicator. It is noteworthy that CM patients with high-risk scores showed marked decreases in immune cell infiltration and compromised immune functions, affecting nearly all cell types. Acknowledging the vital functions of immune cells in antitumor immunity,^[28]^ we speculate that the high-risk CM patients have profoundly suppressed antitumor immune responses. Furthermore, CM patients with high-risk scores exhibited significantly decreased expression of numerous immune checkpoint molecules, except for TNFRSF14, CD276, VTCN1, and TNFSF9. As an immune regulatory protein of cancers, CD276 is overexpressed in melanoma cells and demonstrates an inhibitory role in immune responses.^[30,31]^ TNFRSF14, also named HVEM, is also primarily expressed melanomas, and its inhibiting effects on IFN-γ production contributes to tumor evasion.^[32]^ Similar effects are discovered in VTCN1 and TNFSF9, which is shown to inhibit T cell activation, cytokine production, and the infiltration of regulatory T cells into tumors.^[33–35]^ In conclusion, immune response was definitely suppressed in CM high-risk subgroup, and our study underscored the predictive potential of the lactylation-associated genes risk signature in influencing immune checkpoint expressions and its potential applicability as a reference for CM immunotherapy. Nonetheless, more investigation is warranted to examine the interactions of these genes with immune-related genes.

Although this study identified lncRNAs and hub genes associated with lactylation and hub lactylation-associated gene CALML5 in CM and proposed risk signatures highlighting their potent prognostic value across multiple analyses, it is important for us to acknowledge certain limitations that should be taken into consideration. Firstly, most microarray and clinical CM data used in this study were sourced from publicly-available websites, and thus, our findings need to be validated through additional experimental assays. Secondly, to validate the findings of our retrospective study, additional prospective investigations are warranted. Finally, further investigations are necessary to clarify the precise role and potential mechanisms of hub lactylation-associated genes and lncRNAs in the development of CM.

## 5. Conclusion

In summary, this study has contributed to a better understanding of the functions of lactylation in the pathogenesis of CM. The core lactylation-associated gene CALML5 and hub CALML5-associated lncRNAs MIRLET7BHG, AL162457.2, AL122010.1, MIR193BHG, AC244153.1, AL390760.1, AL133371.2, and CCDC18-AS1 have the potential to serve as biomarkers for the prognosis of CM patients and reflect their immune conditions. To our knowledge, this study was the first bioinformatic analysis examining the role of lactylation in CM, providing a unique perspective for the advancement of therapeutic strategies for CM patients.

## Author contributions

**Conceptualization:** Hailiang Feng.

**Data curation:** Hailiang Feng.

**Formal analysis:** Hailiang Feng.

**Resources:** Hailiang Feng.

**Validation:** Wei Chen.

**Visualization:** Hailiang Feng.

**Writing – original draft:** Hailiang Feng, Chen Zhang.

**Writing – review & editing:** Wei Chen.
